# Highly light-tunable memristors in solution-processed 2D materials/metal composites

**DOI:** 10.1038/s41598-022-23404-5

**Published:** 2022-11-05

**Authors:** Zahra Sheykhifar, Seyed Majid Mohseni

**Affiliations:** Department of Physics, Shahid Beheshti University, Evin, Tehran, 19839 Iran

**Keywords:** Materials science, Electronics, photonics and device physics, Electrical and electronic engineering

## Abstract

Memristors—competitive microelectronic elements which bring together the electronic sensing and memory effects—potentially are able to respond against physical and chemical effects that influence their sensing capability and memory behavior. However, this young topic is still under debate and needs further attention to be highly responding to or remaining intact against physical effects, e.g., light illumination. To contribute to this scenario, using a composite of two-dimensional graphene or MoS_2_ doped with meso-structures of metal/metal-oxides of Ag, Cu and Fe family, we presented scalable and printable memristors. The memristive behavior shows strong dependency upon light illumination with a high record of 10^5^ ON/OFF ratio observed so far in 2-terminal systems based on two-dimensional materials or metal oxide structures. Moreover, we found that the memristors can remain stable without illumination, providing a novel approach to use these composites for developing neuromorphic computing circuits. The sensing and memristive mechanisms are explained based on the electronic properties of the materials. Our introduced materials used in the memristor devices can open new routes to achieve high sensing capability and improve memristance of the future microelectronic elements.

## Introduction

The integration of two-dimensional (2D) materials into electronic circuits gives promise of taking steps beyond the conventional metal-oxide-based technologies^1–4^. Being implemented in microelectronic platforms, e.g., memristors^1,5–11^, such materials with their advanced functionality and outstanding capability circumvent innovative challenges. Memristor device is an electrical resistance switch able to maintain a state of inner resistance based on the history of applied bias and also act both as data storage and information processing elements^1,12–15^. In moving towards the future development of memristors, optical memories have become emerging devices in neuromorphic computing technologies because not only do they directly respond to the optical stimuli, but play a critical role in enhancing the memristive performance^7,10,16,17^. Recently, optical memories based on 2D materials, as the light sensitive centers, have been successfully manufactured in the form of the field-effect transistors (FET) and 2-terminal systems^7,16–18^. Notwithstanding, in spite of all efforts devoted to this field of research, employing 2D materials in microelectronic platforms suffers from a series of limitations including their low-yield device, low speed of processers, fast photocarrier recombination, and large device-to-device variability^6,10,14,19,20^.

It is expected that due to the ultra-thin structure, large specific surface area, and wide detection range^9,17^ of 2D materials such as graphene and molybdenum disulfide (MoS_2_), their doping and sensitization with metal oxides (e.g., iron oxide with its bandgap ranging from insulators to semiconductors)^21^ and active metals (e.g., Ag and Cu) conveys a pathway to achieve the memristive response through driving bias and light illumination^21,22^. In this case, the dominant memristive mechanism originates from the charge trapping/detrapping behavior of effective trapping sites in graphene and MoS_2_^10,17,19,23–25^. Besides, through formation of conductive filaments, oxygen anion and metal cation motions cause the resistance change of the device^6,13^.

Here, using the liquid-phase exfoliated MoS_2_ (or graphene) doped with Fe/Cu (or Ag) nanoparticles (NPs), we designed a two-terminal structure as a highly light-tunable memory. The combination of 2D materials with metal NPs, in addition to enhancing the physical and chemical properties of NPs, also leads to substantial changes in the electrical and electronic properties of 2D materials^26–28^. With intelligent incorporation of 2D materials and metals, the memristors created here achieved the manufacturing multi-functional, highly controllable devices successfully. 2D flakes and metal NPs as both light sensitizer and excitons generation/separation centers can extraordinary improve the photocarriers generation^7,29,30^. In addition, plasmonic metal NPs such as Ag NPs is commonly used to enhance the visible light photoactivity^21,31^. The obtained results in this method are much better than employing two-terminal photonic memristors based on 2D materials or metal oxide reported so far in the literature^7,16,32,33^. By simplifying the circuitry and reducing the power, our devices exhibit a great potential to be employed in energy-efficient optical-sensing based devices; the results are comparable to the current achievements of 2D materials-based memristors. In addition, the manufactured optical memristors can inspire by human brain and visual system, confirming the usefulness of this component in their use in optoelectronic neuromorphic computing systems. This work conveys a pathway toward designing multifunctional memory devices including multilevel storage with superior integration properties for optoelectronic products.

## Results and discussion

**Figure 1:**
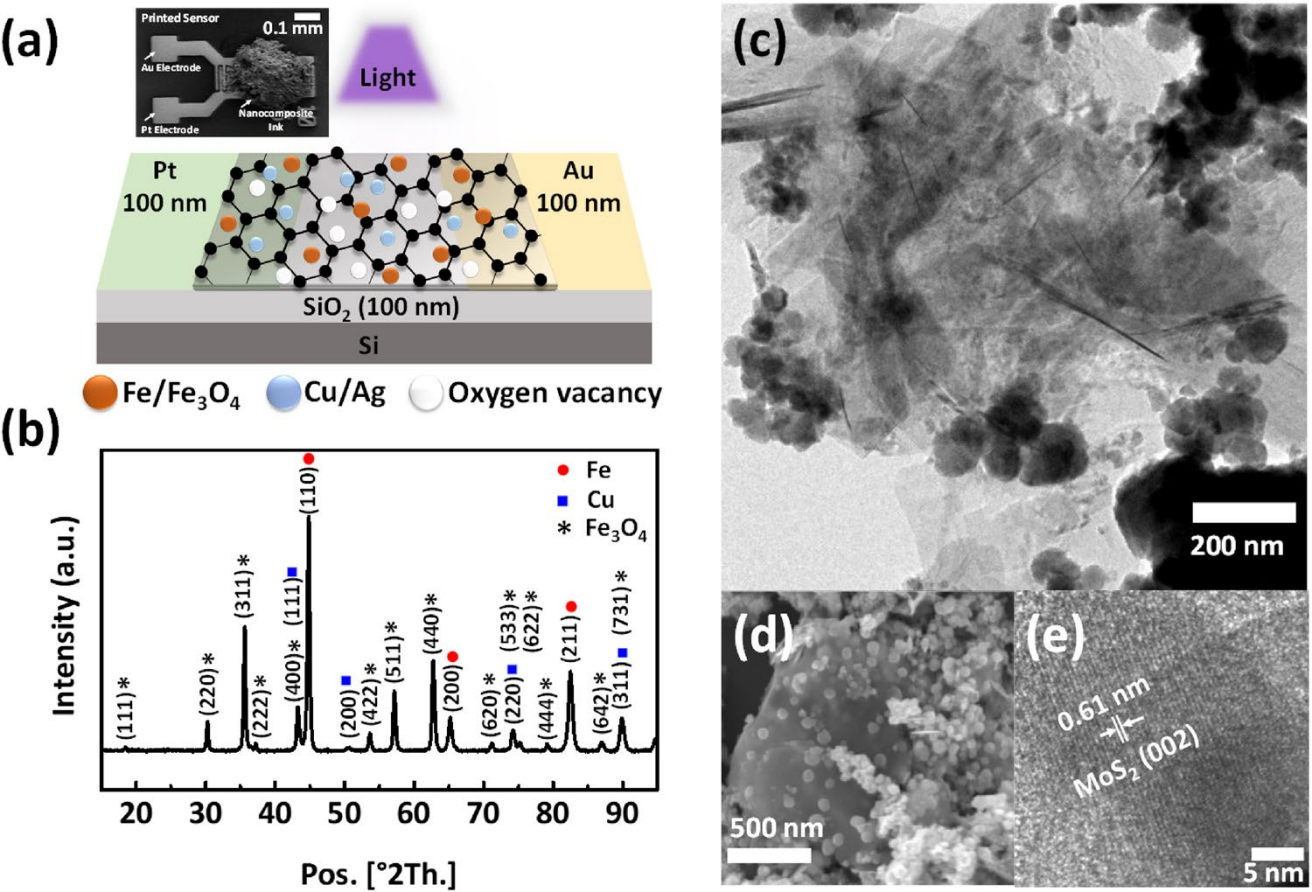
(**a**) Schematic IDE illustrating a two-terminal memristor structure with Au and Pt electrodes. Inset: Top view SEM image of the fabricated device including 2D-material as the light sensitive centers hosting fine degree of NPs. (**b**) XRD patterns of MFC composites. (**c**) and (**d**) TEM and SEM images of MFC composite, respectively. Both TEM and SEM images show that the 2D flakes have been decorated with metallic NPs. (**e**) HRTEM image of the MFC composite. The MoS_2_ crystal could be clearly determined.

As schematically shown in Fig. 1a, our memristor device has been made based on a two-terminal configuration, via an inter-digit electrode (IDE). The distance between the electrodes and the width of an electrode are 5 μm and 10 μm, respectively. The liquid-phase exfoliated MoS_2_ doped with Fe/Cu NPs (MFC) was printed on the top of the IDEs by a dispenser (Digital dispensing valve controller ML-5000X) (see “Methods” section). Inset of Fig. 1a exhibits a top view scanning electron microscopy (SEM) image of the device which included nanocomposite ink with the area of approximately 0.3 mm^2^ and thickness of almost 50 µm. The formation of iron, iron oxide, and copper phase crystallinity is confirmed by the X-Ray diffraction (XRD) pattern of the composite powder, shown in Fig. 1b. No diffraction peaks of well-crystalline MoS_2_ appeared in XRD pattern of sample, indicating that this phase might exist in the form of very small clusters or ultra-thin layers. According to Scherrer relation, the size of copper, iron and iron oxide crystals in the composite based on the MFC are almost 23, 26 and 60 nm, respectively. The morphology of composite examined by transmission electron microscopy (TEM) (shown in Fig. 1c) and SEM (shown in Fig. 1d), displays that MoS_2_ flakes have been decorated by metallic NPs. These NPs have been distributed homogeneously across the whole surface of the flakes and also sandwiched between the flakes during their growth. The flakes are highly transparent and have ultrathin structure. Furthermore, high-resolution TEM (HRTEM) characterizations (Fig. 1e) illustrate that crystal spacing of MoS_2_ element formed as tiny nanocrystals. Further, the information about the size of NPs obtained from the SEM image indicates that the diameter of NPs in MFC-include composite ranges from almost 25 to 65 nm. Accordingly, all the above experimental results can prove being on the right track in developing the successful recipe, resulting in 2D sheets effectively doped with metal NPs.

**Figure 2:**
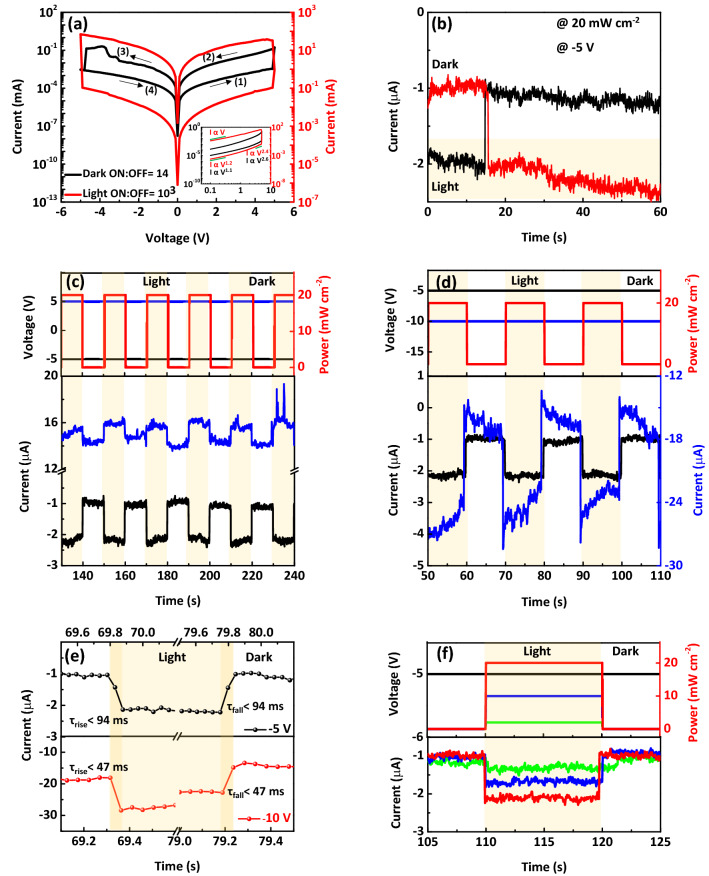
(**a**) I–V curves of sweeps in dark (black line) and light (red line) states for memristor devices based MFC composite. An excellent enhancement of the memristive performance of device was observed under light illumination. Inset: Log–log plot of sweeps from (**a**) which show an Ohmic I–V behavior in the low bias region of both set and reset states. By increasing the bias, the SCLC transport remains the dominant mechanism of device. (**b**) Time evolution of current in − 5 V and the light illumination of 20 mW cm^−2^, denoting the states of programming and erasing with and without illumination, respectively. (**c**) the pulse-switching characteristics of the MFC-based optical memristor under a negative and positive bias. d) The photo-response dynamics of the MFC-based optical memristor under − 5 and − 10 V bias voltages with a power intensity of 20 mW cm^−2^ in three operation cycles. (**e**) The corresponding rise and fall time under − 5 and − 10 V bias voltages. (**f**) Current–Time curve of photo-response as a function of light intensity at – 5 V bias in a single operation cycle.

Switching characteristics were investigated by grounding the Au electrode and sweeping the bias on the Pt electrode with the compliance current of 100 mA at room temperature (RT). The device exhibited a sharp reversible resistive switching when the device conductance increased under a positive biased condition (set state) and vice versa under a negative bias condition (reset state)^34^. Before the light illumination (dark state), the current switching ratio of devices at the read voltage of 0.4 V is 14 and the V_SET_ is 4.9 V, for MFC-based memristor, as shown by black line in Fig. 2a. In stark contrast, under continuous light illumination with a wavelength of 400 nm and a power density of 20 mW cm^−2^, an excellent enhancement of resistive switching performance of devices was observed, as shown by red line in Fig. 2a, indicating the memristor’s improved functionality over the one which is exclusively under electrical operation. The level of progress achieved in the current switching ratio (~ 10^3^) under light illumination of our device is considerably higher than those achieved for two-terminal fabricated memristors based on 2D-material or metal oxide up until now^7,16,32,33^. There is no change in the values of MFC-based memristor devices’ V_SET_ under light illumination compared to the dark state.

To build a better understanding of the origin of the resistance states changes, the logarithmic curve of current–voltage (I–V) has been explored and displayed in inset of Fig. 2a. The slope of the curve at low bias region shows Ohmic conductance. While the sweep at high bias region is described by I α V^α^ (α ~ 2) in dark and light states. This type of I–V dependency is the characteristic of space-charge limited current (SCLC), leading to the charge trapping along the conductive filament path^13,35–37^. Observation of SCLC transport in MFC-based memristor device is due to the existence of sulfur vacancy (V_S_) as well as oxygen vacancies (V_o_) and Cu ions motions, influencing the memristivity^8,35,38^. In addition, 2D materials provide charge trapping centers for photo-generated charge carriers (electrons/holes), and store the photo-generated electrons/holes even after the removal of light stimuli resulting in a significant improvement in the memristive performance^7,19^.

The MFC-based optical memristor is exposed to the light of 20 mW cm^−2^ after 15 s under − 5 V bias (as shown in red plot in Fig. 2b), indicating a sharply photo-response dynamics, where the photogenerated charge carriers in the MoS_2_ can change the resistance states that would affect the device performance. The current value is stable for at least 45 s as long as the light is illuminated, which resulted in the difference between the light and dark states. Our device was also examined in inverse conditions, when was removed the light illumination after 15 s (as shown in black plot in Fig. 2b), confirming well the repeatability of both resistance values with and without illumination.

Figure 2c presents the optical pulse-switching characteristics of the MFC-based optical memristor under a constant negative and positive bias, and no performance degradation was observed. A larger photocurrent was obtained at − 5 V than 5 V bias, caused by the built-in electric field^39^. The current enhancement was operated by an optical pulse with a power intensity of 20 mW cm^−2^ and a pulse width of 10 s. The reversal process was initiated by removing the light illumination. The optical memory can be repetitive switched between two resistance states and delivers the current switching ratio of ~ 2. It should be noted here that the performance progress of the MFC-based optical memristor after illumination is less compared to what mentioned in Fig. 1a, which is likely due to the increased photogenerated charge recombination at a short interval time.

Figure 2d shows the photo-response dynamics of the MFC-based optical memristor at different biases with a power intensity of 20 mW cm^−2^ for optical pulse. The current of dark and light states reached the steady state condition faster at − 5 V bias, while another mode (i.e., − 10 V bias) take more time to relax. The rise time (τ_rise_) and fall time (τ_fall_) of the MFC-based optical memristor were estimated at different biases. The τ_rise_ is defined as the time required by the optical memory to increase its photocurrent value from 10 to 90% of the peak value while τ_fall_ is just the opposite (i.e., the time from 90 to 10%)^40^. As shown in Fig. 2e, it was observed that τ_rise_ and τ_fall_ to be less than 47 ms and 94 ms under − 5 V and − 10 V biases, respectively. Appling a higher bias resulted in faster photo-response compared to − 5 V bias, which was mainly attributed to faster charge separation and more efficient carrier transport at interfaces^39^.

Current–Time (I–t) curve of photo-response as a function of light intensity at − 5 V bias was investigated as shown in Fig. 2f. All the plots exhibited a sensitive and reproducible photo-response. When the irradiation power was raised from 2 to 10 and 20 mW cm^−2^, a continuously increased photocurrent was observed, followed by stabilization at around 1.4, 1.7 and 2.2 µA, respectively. As compared to previous reports, the photo-response time of our optical memristor is much shorter by two orders than that of all 2D material heterostructure-based optical memories, indicating a promising light-switching behavior of the as-prepared devices^39,41–43^.

**Figure 3:**
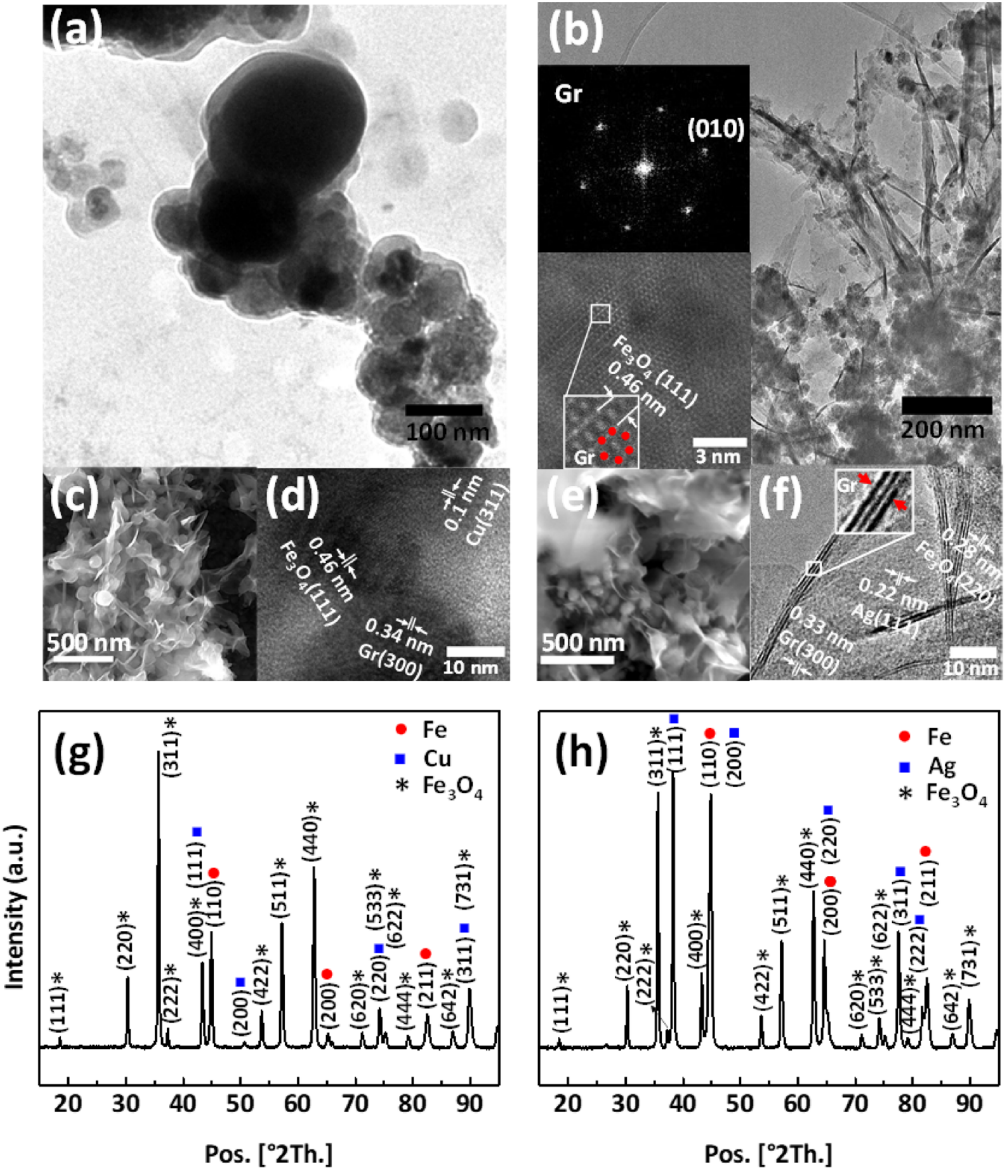
(**a, b**) TEM images of composites including GFC (**a**) and GFA (**b**) composites. The TEM images show that the 2D flakes have been decorated with metallic NPs. Fast Fourier transform in (**b**) (the top left inset) shows the graphene crystal in GFA composite. The bottom left image in (**b**) also exhibits that the sheets have four layers, confirming an ultrathin morphology of graphene. (**c**)The FESEM and (**d**) HRTEM images of the GFC composite. (**e**) The SEM and (**f**) HRTEM images of the GFA composite. The SEM/FESEM images confirm the TEM results. The graphene (Gr), Ag, Cu, Fe, and Fe_3_O_4_ crystals could be clearly determined in the HRTEM images. (**g, h**) XRD patterns of composites, including GFC (**g**) and GFA (**h**) composites. All two plots illustrate metal and metal oxide phases which had been confirmed by HRTEM results.

In continue, we replaced MoS_2_ with graphene to compare the performance of MFC-based optical memristor with the other 2D materials. In the other hands, silver NPs have an absorption at UV wavelength range and it is also more conductive than cupper NPs. Therefore, replacing silver with cupper not only enhances the memristic property, but also enhances its response to UV light illumination. The liquid-phase exfoliated graphene doped with Fe/Cu and Fe/Ag NPs are called GFC and GFA, respectively. The TEM images in Fig. 3a,b present the morphology of GFC and GFA nanocomposite structures, respectively. It was clearly observed that the highly transparent layers of graphene have ultrathin structure and the NPs are placed in between them or randomly distributed across the whole surface of 2D sheets. SEM/FESEM images, also, confirm TEM results as shown in Fig. 3c,e, including GFC (c) and GFA (e) composites. Furter, the information about the size of NPs obtained from the SEM/FESEM images indicates that the diameter of NPs in GFC- and GFA-include composite ranges from almost 55 to 120 nm and 45 to 110 nm, respectively. As shown in Fig. 3d,f, HRTEM images represent that crystal spacing of various elements formed, is in excellent agreement with the appearance of facets, which is in turn consistent with the X-ray diffraction (XRD) results (Fig. 3g,h). Fast Fourier transform (FFT) pattern in the top inset of Fig. 3b confirms the hexagonal arrangement of C elements in graphene layers for GFA composite. Doping graphene sheets with NPs can be detected in the bottom inset of Fig. 3b. The red dots in this figure shows an obvious hexagonal lattice which is a direct evidence of the existence of graphene phase doped with iron oxide NPs. The layer number of graphene is less than four, confirming the existence of layered ultrathin sheets as shown in Fig. 3f. The formation of iron, iron oxide, copper, and silver phase crystallinity is confirmed by the XRD patterns of the composites powder, shown in Fig. 3g,h. According to Scherrer relation, the size of copper, iron, and iron oxide crystals in the composite based on the GFC are 32, 30 and 65 nm, respectively. However, the size of silver, iron and iron oxide crystals in the GFA sample are 36, 33, and 60 nm, respectively. Accordingly, all the above experimental results indicating that consistent high-quality nanocomposite can be achieved using our synthesis method.

**Figure 4:**
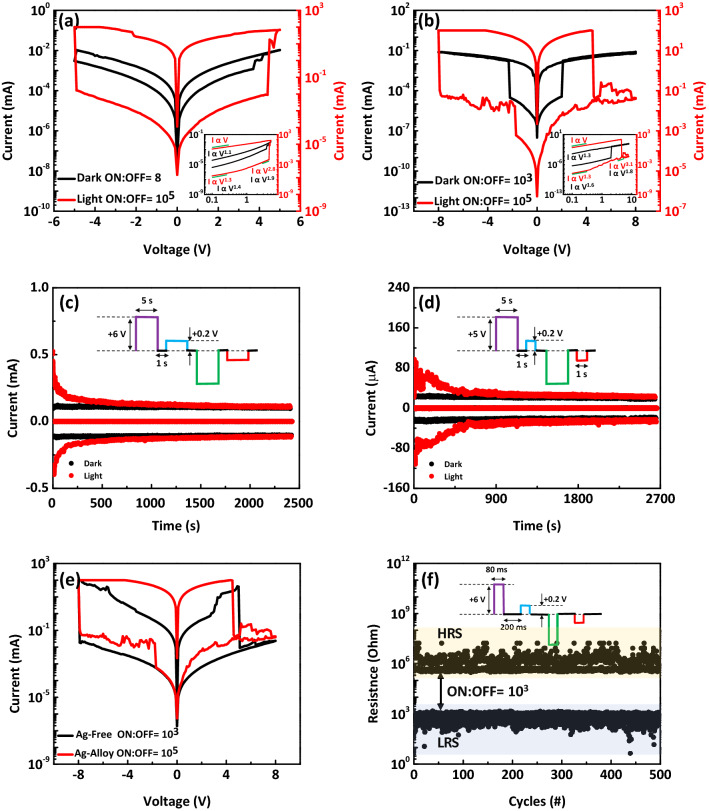
(**a, b**) I–V curves of sweeps in dark (black plot) and light (red plot) states for memristor devices based on GFC (**a**) and GFA (**b**) composites. An excellent enhancement of the memristive performance of devices was observed under light illumination. Inset: Log–log plot of sweeps which show an Ohmic I–V behavior in the low bias region of both set and reset states. In the dark state (black plots), by increasing the bias, the SCLC transport remains the dominant mechanism of graphene-include devices. However, due to the TCLC transport, the slope of the curves has increased under light illumination (red plots). (**c, d**) Symmetric switching performance test of on GFC (**c**) and GFA (**d**) composites for one hundred cycles in dark (black plot) and light (red plot) states. The inset shows the shape of the applied voltage pulse, which consists of four segments. (**e**) The electrical I–V characteristic of active metal-free device (black plot) and GFA-based optical memristor (red plot) under light illumination with 20 mW cm^−2^. (**f**) Time-dependent measurement of GFA-based optical memristor switch featuring stable endurance over 600 cycles in dark state. The resistance of the high resistance state (HRS) and low resistance state (LRS) is determined by measuring the current at a small bias of 0.2 V.

Electrical I–V characteristics were measured using bipolar DC voltage sweeps, exhibiting a sharp reversible resistive switching for both dark and light states, as shown in Fig. 4a,b for GFC- and GFA-based devices, respectively. The voltage was applied to Pt electrode while the Au electrode was grounded at room temperature. The SET and RESET transitions occur at positive and negative voltages, respectively. The current switching ratio read at 0.4 V before the light illumination (dark state) is 8 and 10^3^ for GFC- and GFA-based optical memristors, respectively, indicating significantly high device performance using Ag NPs as active metal. In dark state, the value of V_SET_ is 2 V for GFA-based memristor, which is smaller than that of GFC-based memristors (3.7 V). Therefore, the heterostructure of graphene-Fe/Ag ensures obtaining low operating voltage to observe resistive switching behavior in this memory device. In stark contrast, under continuous light illumination with a wavelength of 400 nm and a power density of 20 mW cm^−2^, an excellent enhancement of resistive switching performance of devices was observed, as shown by red line in Fig. 4a, b, indicating the memristor’s improved functionality over the one which is exclusively under electrical operation. The graphene-include memristor devices (i.e., GFC- and GFA-based memristor devices) show ON/OFF ratio of ~ 10^5^ which is one hundred times larger than that of MFC-based memristor device (~ 10^3^); this observation could be due to the unique gapless band structure of graphene and its superior carrier mobility resulting in giving a better response to light illumination^19,44^. The level of progress achieved in the current switching ratio under light illumination of our devices is considerably higher than those achieved for two-terminal fabricated memristors based on 2D-material or metal oxide up until now^7,16,32,33^. The values of V_SET_ under light illumination are 4.4 V and 4.5 V for GFC- and GFA-based memristors, respectively, which are higher than those of only under electrical set operation, suggesting the increase of the number of the traps and hence the trapped charges^45,46^. In light state, the current of the low resistance state reaches 100 mA, which is due to the high conductivity of the conductive copper or silver filaments. To control it, the distance between the contacts can be increased so that there is less current in the system. In addition, the area through which the current passes is larger compared to the areas used in the devices of the previous articles leading to high charge flux observed in the device of the current study.

The logarithmic curve of current–voltage (I–V) is displayed in insets of Fig. 4a,b for GFC- and GFA-based memristors, respectively. The slope of the curve at low bias region shows Ohmic conductance. While the sweep at high bias region is dependent on SCLC, leading to the charge trapping along the conductive filament path^13,35,36,47^. As shown by red plots in insets of Fig. 4a,b, in graphene-include devices, the slope of the curve has increased under light illumination (I α V^α^ (α˃2)); the memristive mechanism of SCLC is strong or is the same as trapped-charge limited current (TCLC)^36,48,49^. This could be explained by the increased free charge carrier concentration^7^. In this condition, 2D materials provide charge trapping centers for photo-generated charge carriers (electrons/holes), and store the photo-generated electrons/holes even after the removal of light stimuli resulting in a significant improvement in the memristive performance^7,19,50^.

The pulse cycling test for practical applications is an important matter that allow us to mimic the basic features of synaptic plasticity in emulating the learning and memory functions of the human brain. Our device resistance states can also be continuously tuned by applying voltage pulses in both dark and light states. Figure 4c,d illustrate data retention and symmetric switching using wider bipolar voltage pulses with over one hundred switching cycles for GFC- and GFA-based optical memristors, respectively. The voltage pulse including of 5 s width with + 5 V for set, − 5 V for reset and 1 s width with 0.2 V for reading bias is schematically presented in inset of Fig. 4c,d. After 2.7*10^3^ s, the device current due to positive and negative programming pulses exhibits significant degradation in light state. In stark contrast, there are any clearly change in device current even after this time in the absence of light illumination. These data reflect high robustness, an excellent controllability over cycle-to-cycle consistency, and great stability, which has always been a major concern in the memory industry.

To build a better understanding of the role of active metal in our optical memristor devices, an Ag (or Cu)-free NCs is prepared using only graphene and Fe NPs. Figure 4e exhibits a significant variation in electrical I–V characteristic of active metal-free device compered to GFA-based optical memristor under light illumination with 20 mW cm^−2^. The current switching ratio in Ag-free device is considerably less than that in Ag include-based optical memristor, indicating Ag NPs as a possible plasmonic metal NPs can sharply enhance the visible light photoactivity in addition to the grows of conductive filaments^21,31^. In Ag-free composite, the electron–hole pairs would be generated when graphene and iron-oxide semiconductor absorbed the light with energy larger than their bandgap^51,52^, resulting in enhancement of memristive performance under light illumination compared to the dark state. Figure 4f represents the endurance characteristic of GFA-based optical memristor during 700 s at dark state. The voltage pulse including of 80 ms width with + 6 V for set, − 6 V for reset and 200 ms width with 0.2 V for reading bias schematically shown in inset of Fig. 4f. The current switching ratio 10^3^ is remained for over 500 cycles, where a clear resistance window can be seen. The obtained data encourages us for making the synaptic devices using silver NPs with higher performance compered of copper NPs at dark state.

**Figure 5:**
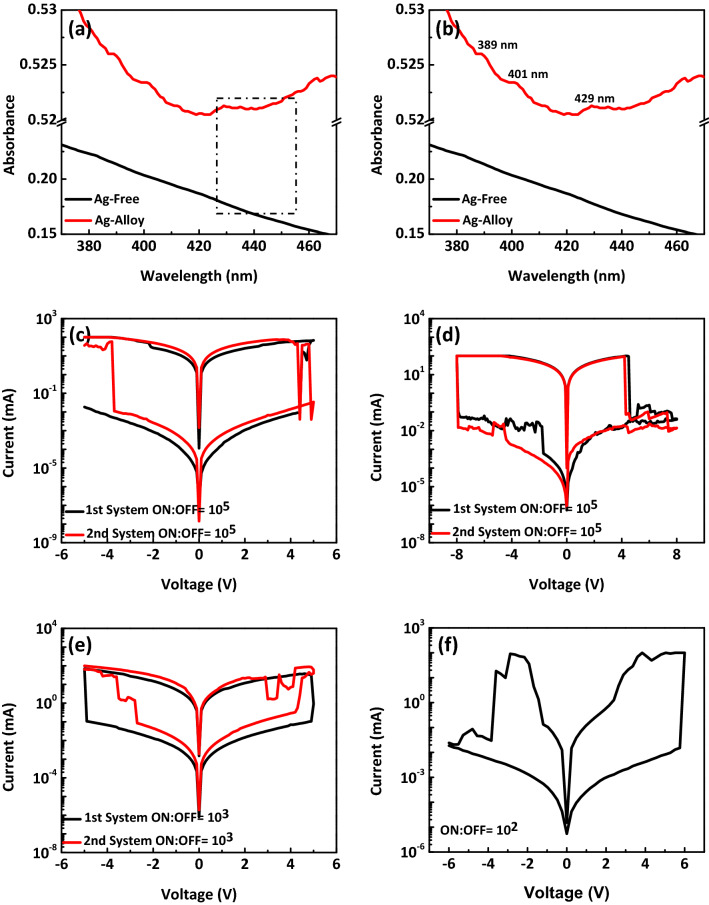
(**a**) UV–vis spectra of GFA-include (red plot) and GF-include (black plot) composite. (**b**) The absorbance spectra of the specified range displayed by the dashed line in (**a**). (**c**–**e**) I–V curves of sweeps for the fabricated second memristor devices based on GFC (**c**), GFA (**d**) and MFC (**e**) composites. (**f**) The switching characteristic for the MFC-based memristor device that has been made based on a new IDE.

As shown in Fig. 5a, the UV–Vis spectra were measured to characterize optical properties of GFA and GF-include composites, indicating enhancement of UV light absorption by using Ag NPs. Further, to be able to make comparison between Ag-free and Ag-include composites, the readers are provided with the absorbance spectra of the specified range displayed by the dashed line. It is obvious that the absorbance spectrum of GFA-include composite demonstrates the typical surface plasmon resonance (SPR) bands of Ag nanoparticles with three peaks at about 389, 401 and 429 nm (Fig. 5b). With light illumination, in addition to improving the performance of the made optical memristor systems, the conductivity of the high resistance state in the light state should also increase compared to the conductivity of the high resistance state in the dark state. For example, in voltage 0.4 V, the current of high resistance state under light illumination are 90 nA, 31 nA and 1 µA for GFA-, GFC- and MFC-based optical memristors, respectively, which is greater than their values in the dark state, i.e. 2, 21 and 46 nA for GFA-, GFC- and MFC-based optical memristors, respectively (as shown in Figs. 2a, 4a,b)). To ensure about performance reproducibility, many memristor devices were made and they have shown working parameters variation within a small range. Some of those memristors were tested against optical effects under light illumination. The measurement results are obtained for another series of re-production systems fabricated under similar conditions. As shown in the Fig. 5c–e the on/off ratio of all three optical memristor systems based on GFC-, GFA-, and MFC-include optical memristors are 10^5^, 10^5^, and 10^3^, respectively, each of which does not show any noticeable change compared to those of the first system. Figure 5f shows the switching characteristic for the MFC-based memristor device that has been made based on a new IDE. In this case, the distance between the electrodes and the width of an electrode are 10 μm. The on/off ratio is 10^2^ that is lower than earlier system (Fig. 2a), indicating the presented memristor is scalable and printable. Compared to vertically stacked structure which most of the memristor devices utilize, our memristors using the IDE structure have higher operation voltage. Indeed, in the vertical structure, due to the small distance between the electrodes (a few nanometers), the threshold voltage is also lower. By increasing the distance between the electrodes through IDE, the higher operation voltage is needed.

**Figure 6:**
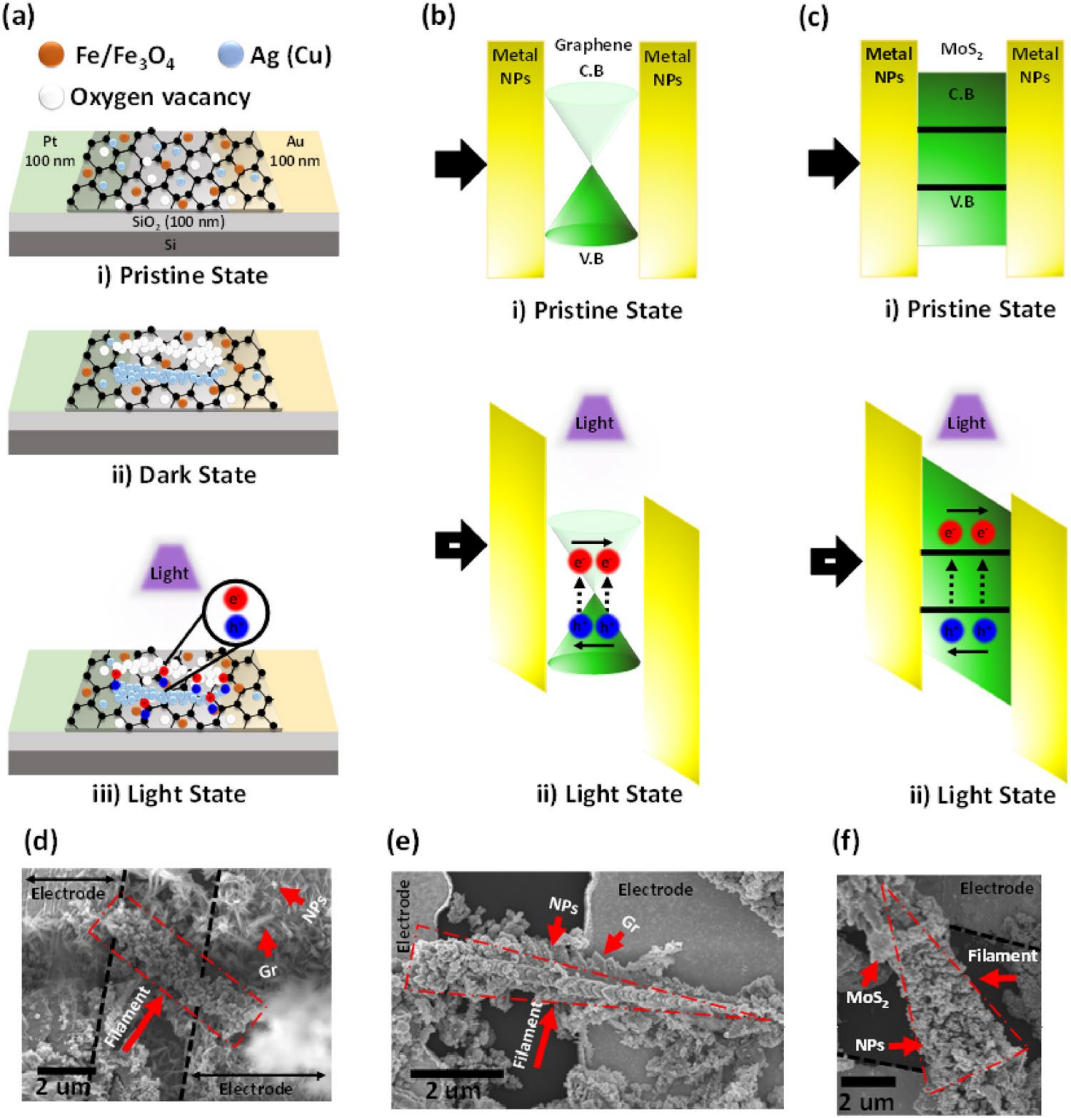
Schematic diagrams of the switching performance corresponding to the various states of a 2D materials-based memristor device doped with NPs, schematic energy band diagram in dark state and under light illumination and SEM images. (**a**) (i) The pristine state in which the device is in high resistance state (OFF state). A random distribution of NPs on the 2D flakes can be observed. (ii) The set state (ON state) occurs when an electrically conductive path is formed within the channels. (iii) Generation of electrons and holes in storage medium by photon absorption. (**b**) Schematic energy band diagram of graphene-include memristor device at i) initial state and ii) under light illumination. (**c**) Schematic energy band diagram of MoS_2_-include memristor device at (i) initial state and (ii) under light illumination. (**d**–**f**) SEM images from Ag and Cu filaments as electrical conductive paths in devices which include graphene-Fe/Ag (**d**), graphene-Fe/Cu (**e**) and MoS_2_–Fe/Cu (**f**) composites, confirming the proposed model.

We present a fundamental mechanism to support the experimental achievements. As shown in Fig. 6a, there are random distribution of metal and metal oxide NPs within the 2D flakes in step i, the initial state, (as earlier confirmed in TEM and SEM images). In dark state, the negative biased Pt electrode attracts oxygen vacancies and cations in the storage medium to form filaments with a high electrical conductivity^4,6,23,24,53,54^. The device switches to ON state whenever one or more conductive filaments are formed (step ii). A voltage with the reverse polarity is applied to switch the device to OFF. Applying positive bias to the Pt electrode drives the oxygen vacancies away from the electrode and tears up the conductive filaments. Furthermore, under light illumination, the carriers transition is attributed to the charge trapping/detrapping behavior in graphene and MoS_2_ which are effective trapping sites^10,17,19,23–25,37^. Photovoltaic effect initially creates excitons in such 2D materials by photon absorption with energy higher than their band gap (step iii). By applying an external electrical field, the photogenerated excitons are separated in the 2D material-NPs interface and move toward the electrodes in opposite direction^55^. The more the number of the photogenerated free charge carriers increases, the more the bending degree of energy band increases, leading to the reduction of the potential barrier height and, thus, resulting in a larger current^7,25,56–58^, as schematically illustrated in Fig. 6b,c. The reset process is similar to that of the electrical switching in dark condition, as stated above. Moreover, in the reset process, the positive biased Pt electrode attracts the trapped electrons from the trapping sites in 2D materials, leading to the further increase of the potential barrier height and recovering the original state (i.e., HRS)^7,17^. The SEM images shown in Fig. 6d–f confirm the formation of Cu (Ag) filaments when the devices are in set state. These filaments can easily grow to a few microns, whereas the growth of filaments has been usually reported to be at the level of nanometers in the previous studies. To demonstrate the movement of anions and cations as the result of applying bias, we fabricated a disk of MFC-include composite powder that it has not been shown here. Conducting systematic EDS mappings of the points under the probe, before and after biasing confirmed the existence of the inhomogeneous distribution of Cu and O in the pristine state. In contrast, the decrease of the amount of Cu and the increase of the amount of oxygen observed at the point in which the positive bias has been applied, suggest that applying electric field makes O ions migrate towards the positive electrode while Cu cations diffuse in the opposite direction.

## Conclusions

In conclusion, we demonstrated that our 2D-materials-based memristor devices are light-tunable with a high ON/OFF ratio at room temperature. The significant improvement in memristive performance was observed under light illumination, which thus far has not been observed in the previous 2-terminal memristor devices based on 2D-materials or metal oxide structures. Depending on NPs and 2D-materials type in the storage medium, the response of the devices in dark and light states shows difference, which makes it possible to engineer these devices in accordance with their various performance requirements. Graphene (or MoS_2_) combined with copper produces almost low performance memristive response without illumination. Nevertheless, replacing it by silver results in a large switching variation in such devices. The obtained pulse results allow us to mimic the basic features of synaptic plasticity in emulating the learning and memory functions of the human brain. The integration of light-sensitive systems, with improved performance in memristive against the external changes of light illumination, is considered a remarkable progress, and thus, holds great promise application prospects in memristive systems and neuromorphic computing technologies.

## Methods

### Memristor devices fabrication

By means of wet oxidation, a 100-nm-thick SiO_2_ layer was deposited on the top of the Si wafer. The standard photolithography and lift-off processes were employed to pattern Pt and Au electrodes. The 100-nm-thick Pt and Au electrodes were e-beam evaporated.

### Composite fabrication

The Graphene (MoS_2_) layers were prepared by one-step and simultaneous exfoliation-electrodeposition method in an ionic solution of FeSO_4_.7H_2_O (0.1 M), CuSO_4_.5H_2_O (Ag_2_SO_4_) (0.002 M), and SDS (10^−2^ g) (98.0% Merck) at room temperature. Controlling the electrode potentials results in the alternate exfoliation of 2D sheets and deposition of metal NPs on the layered sheets. Platinum and Graphite (MoS_2_ bulk) were used as cathode and anode electrodes, respectively. After 12 cycles of AC current (1 cycle (I/t): 250-mA/120-s, 40-mA/10-s, 2-mA/40-s), the achieved product is collected by a magnet from the solution, washed by double deionized water and, at the end, dispersed in deionized water. Finally, the composite ink has been printed on the fabricated inter-digit electrode by despising.

### Device characterization

The top-view image of the device and imaging of the composites were obtained by using scanning electron microscope (SEM) and Field emission SEM (FESEM). The imaging of composites was conducted by making use of the tunneling electron microscopy (TEM) as well as high resolution TEM (HRTEM). The crystallinity of NPs confirms using the X-ray diffraction (XRD) patterns. The electrical measurements were conducted by employing a semiconductor parameter analyzer and Keithley-2613 for DC and pulsi measurements, respectively. Electrical bias was applied to the Pt electrode while the Au electrode was grounded.

## Data Availability

The data supporting the findings of this study are available from the corresponding author upon request.
